# Simulation-based training in asthma exacerbation for medical students: effect of prior exposure to simulation training on performance

**DOI:** 10.1186/s12909-022-03300-2

**Published:** 2022-03-31

**Authors:** Zhenhua Liu, Qiong Chen, Jing Wu, Xinhua Li, Yuchen He, Qiao Yu

**Affiliations:** 1 Department of Neurology, Xiangya Hospital of Central South University, Changsha, 410008 Hunan China; 2 Department of Gerontology and Respirology, Xiangya Hospital of Central South University, Changsha, 410008 Hunan China; 3 National Clinical Research Center for Geriatric Disorders, Xiangya Hospital of Central South University, Changsha, 410008 Hunan China; 4 Clinical Skills Training Center, Xiangya Hospital of Central South University, Changsha, 410008 Hunan China; 5 Department of Internal Medicine, Xiangya Hospital of Central South University, Changsha, 410008 Hunan China; 6 Medical Virtual Reality Research Center of Central South University, Changsha, 410008 Hunan China

**Keywords:** Simulation, Simulation-based training, Asthma exacerbation, Medical student, Simian@3G, Communication, Medical humanistic care

## Abstract

**Objectives:**

To evaluate the effect of prior exposure to simulation-based training on medical students’ performance in simulation-based training in asthma exacerbation.

**Methods:**

Third-year novice medical students, who had no prior experience in simulation education and completed diagnostics and internal medicine courses, were recruited in this prospective observational study and divided into the pre-exposure and unexposed groups. Each group received a four-hour asthma exacerbation simulation-based training. The pre-exposure group was exposed to a myocardial infarction simulation training two weeks prior to the asthma simulation training. The main outcome was the performance scores in clinical skills and abilities. Performance and task checklist completion were recorded during the training. In addition, the knowledge level was tested before and after the simulation training. Students’ satisfaction was evaluated using a feedback questionnaire.

**Results:**

In a class of 203 third-year novice medical students, 101 (49.8%) and 102 (50.2%) were assigned to the unexposed and pre-exposure groups, respectively. Scores were higher in the post-simulation test compared with the pre-simulation test. Checklist completion was greater in the pre-exposure group compared with the unexposed group (*p* < 0.001). Performances in communication and medical humanistic care were better in the pre-exposure group than in the unexposed group (*p* < 0.001). There were no differences in medical history taking, physical examination, auxiliary examination interpretation and treatment formulation between the two groups (*p* > 0.001). Totally 73.21% and 26.13% of students strongly agreed and agreed, respectively, that asthma exacerbation simulation-based training was necessary and valuable.

**Conclusions:**

Prior exposure to simulation training can improve performance in medical students, including communication skills, medical humanistic care and checklist completion in subsequent asthma exacerbation simulation-based training.

**Supplementary Information:**

The online version contains supplementary material available at 10.1186/s12909-022-03300-2.

## Background

Simulation-based training (SBT) is an educational method used for environment-controlled, team-based proactive and hands-on learning. Students can work in an immersive and realistic way to learn a variety of clinical skills without compromising patient outcomes [1]. High-fidelity simulation uses a computer-controlled 3D simulator for displaying various clinical manifestations, while an experienced instructor manages the mannequin according to the simulated case. SBT is considered an efficient tool for teaching competency tasks, including professionalism, medical knowledge, team performance, medical humanistic care and communications skills [2, 3]. The advantages of high-fidelity simulation have been demonstrated in several studies in critical care, emergency medicine, surgery and anesthesiology [4, 5]. Simulation allows task repetition and gradual increase in complexity, which provides students with an opportunity for reflection, effective feedback and debriefing [6, 7].

The most widely adopted pedagogical approaches involve traditional teaching methods, for example, lectures to foster knowledge and small group training for novices to gain proficiency in clinical skills. Asthma exacerbation is frequently encountered in the emergency department. Early detection and treatment of asthma exacerbation is crucial to preventing disease progression [8]. However, medical students have few opportunities to learn from real and critically ill asthma cases. For patient safety, medical students should not manage an asthma exacerbation, for the first time, in patients. SBT with high fidelity simulator provides a reasonable educational opportunity for medical students to gain experience in managing an emergency with these severely ill patients.

As mentioned in previous reports, there is no doubt about the benefits of SBT in learning asthma exacerbation, but whether more simulation-based training would be better is unknown [3, 6, 9]. It was demonstrated that students progress rapidly and acquire higher performance scores after practicing with different scenarios or repeatedly practicing on the same scenario [10]. However, due to limited teaching resources, many students do not have the opportunity to perform a scenario simulation more than once. A professional subject is often equipped with only one simulation course per scene. It is unclear whether only one scenario simulation training per subject would be helpful for students to follow-up scenario simulation learning in other subjects. We proposed the hypothesis that prior exposure to SBT would improve the performance of medical students in the subsequent SBT. The purpose of this study was to evaluate the effect of previous exposure to SBT on knowledge improvement, clinical skills, communication skills and humanistic care, in subsequent SBT.

## Methods

### Study design and participants

This was a prospective observational study to evaluate the effect of previous SBT experience on performance in following asthma exacerbation SBT. The study was performed in Clinical Skills Training Center from September 2020 to January 2021 in Xiangya Hospital of Central South University, and was approved by the Ethics Committee of the Xiangya Hospital of Central South University.

A total of 203 medical students were recruited as participants. All medical students are undergraduates of 2017 grade from Xiangya Medical School, Central South University. Inclusion criteria were: (1) third-year medical students and aged > 18 years; (2) completion of diagnostics and internal medicine courses; (3) no prior experience of simulation education; (4) consent to participate in “Asthma Simulation Course”. The participants were grouped into the pre-exposure and unexposed groups by student number, i.e., no complete randomization. Grouping by student number is the most common and convenient grouping method in our medical school. One group was exposed to myocardial infarction scenario two weeks prior to the asthma simulation [11], constituting the “pre-exposure group”. The other group was not exposed to other scenarios before receiving the asthma simulation, constituting the “unexposed group”. Each group was divided into 25 subgroups of 4 to 5 medical students. Students in each subgroup were selected by lots to play the roles of a doctor, a physician assistant, a nurse, and family members (1–2 students).

### Pre-simulation quiz

Each student completed a pre-simulation self-assessment quiz, which comprised 10 multiple-choice questions pertaining to the pathophysiology, diagnosis and treatment of asthma.

### Simulation flowchart

**Fig. 1:**
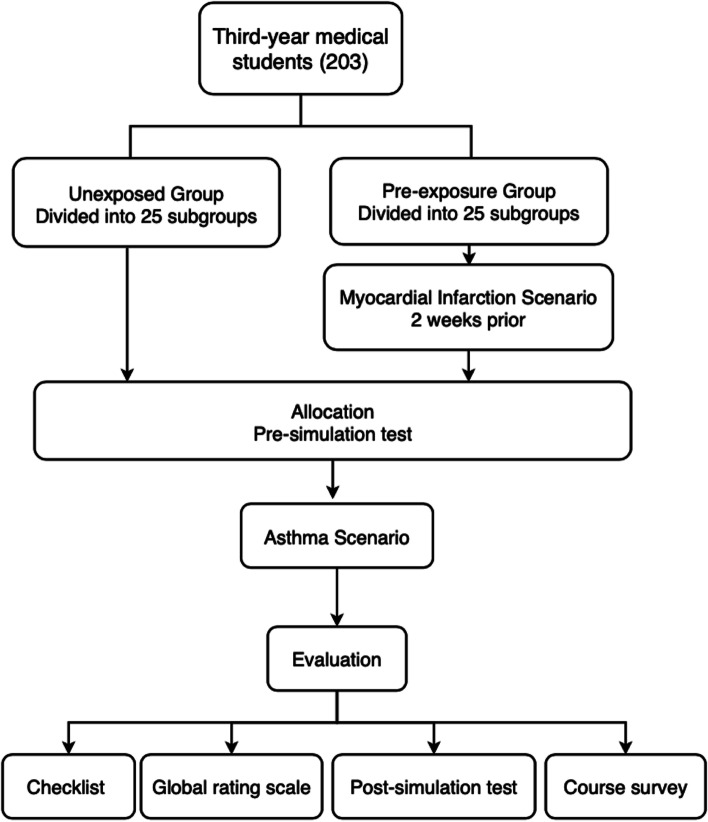
Study design

After quiz completion, the participants were introduced and oriented to Simian@3G by a simulation training specialist who examined the patient simulator and set up a scenario during simulation training (Fig. 1).

The myocardial infarction scenario was ST segment Elevation Myocardial Infarction (STEMI) in the inferior wall occurring in the emergency department. The simulation performance ran for approximately 10 min. If no correct treatment was administered, the case culminated in cardiopulmonary arrest; in case the critical action and treatment were completed, the simulation ended before the 10-min mark (Supplement to Fig. 1).

**Fig. 2:**
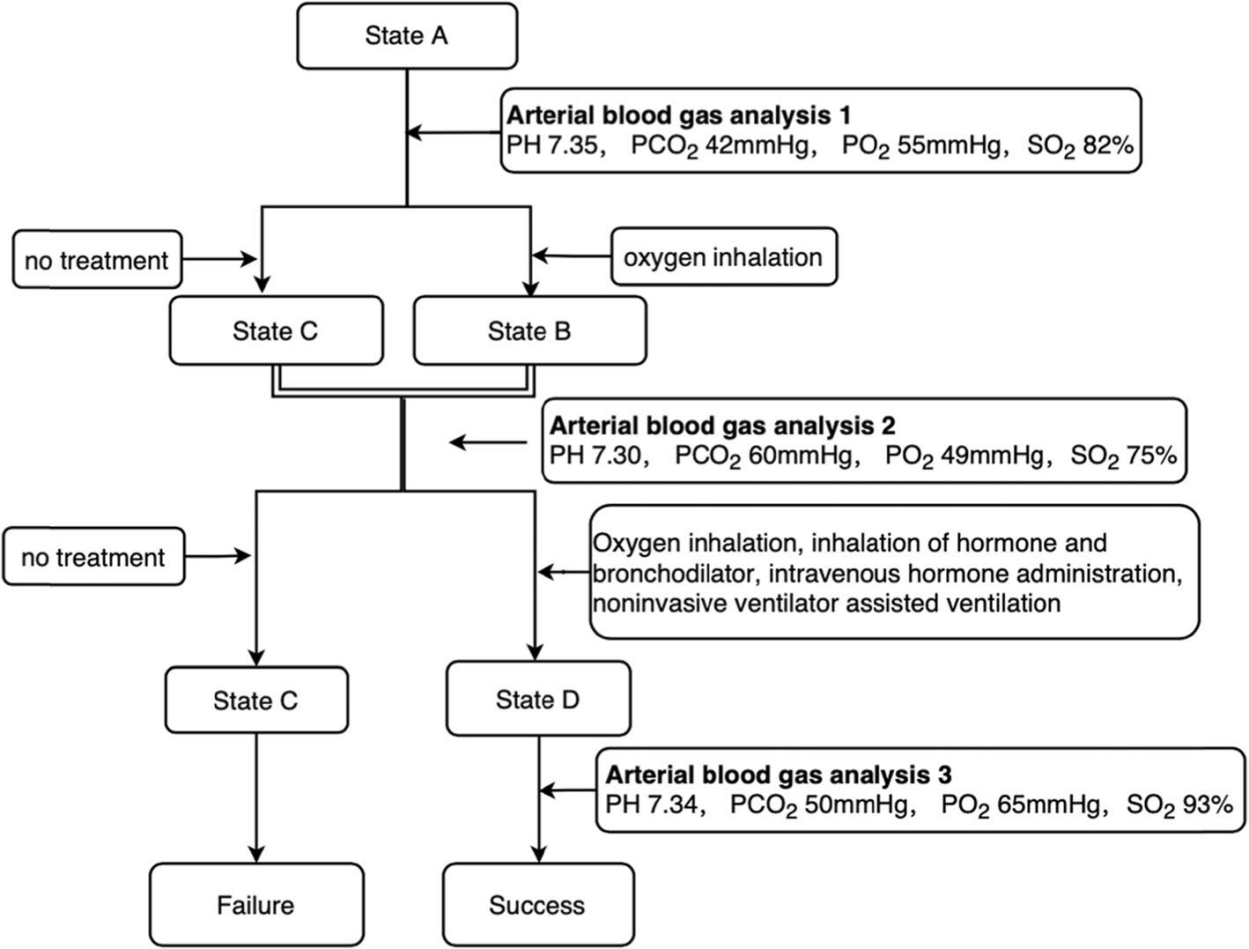
Scenario flow chart of asthma exacerbation. Annotations: **A**, **B**, **C** and **D** present states of simulated patient. State **A**: Conscious, dyspnea, wheezing during expiratory phase of breathing,HR 110 bpm, RR 30bpb, Bp135/80 mmHg Oxygen saturation (SaO2) 91%. State **B**: Conscious, exacerbated dyspnea and wheezing, HR 110 bpm, RR 32bpb, Bp160/90 mmHg, SaO2 85%. State **C**: Conscious, exacerbated dyspnea and wheezing, HR 110 bpm, RR 32bpb, Bp160/90 mmHg, SaO2 75%. State **D**: Conscious, exacerbated dyspnea and wheezing, HR 100 bpm, RR 28bpb, Bp130/80 mmHg, SaO2 95%

The study scenario involved a simulation of a moderate asthma exacerbation occurring in the emergency department. The simulation performance ran for approximately 10 min. If no correct treatment was administered, the case culminated in cardiopulmonary arrest; in case the critical action and treatment were completed, the simulation ended before the 10-min mark (Fig. 2).

Participants were clearly instructed to express their thought processes and actions ("think aloud") and to employ teamwork skills, including leadership, role delegation, mutual respect and closed-loop communication. During each simulation, two independent assessors, who were usually seasoned senior clinicians and experienced in simulation evaluation, observed the entire training process and scored the performance of each group in the management of asthma exacerbation. Assessors were blinded to group allocation.

### Checklist

A checklist was used to record the achievements of the simulation and to evaluate participants during simulation. The checklist was based on previous reports [12, 13] and adjusted according to our specific case and discussion with experts in respiratory medicine. There were 22 items in the checklist (Supplementary Table 1), which were graded with a binary rating (“achieved” or “not achieved”), including medical history taking (6 items), physical examination (7 items), observation of disease development (3 items), auxiliary examination interpretation (2 items), treatment (3 items) and diagnosis (1 item).

### Global rating scales

We next used the global rating scale (GRS) to assess clinical competency. The GRS typically requires assessors to rate the overall performance of participants based on expert judgment. Five-point Likert scales were used in rating scales to evaluate the performance of participants. There were six items in the rating scales, including medical history taking, physical examination, auxiliary examination interpretation, treatment, communication skills and medical humanistic care, with the last two being the basis of medicine throughout history. Good communication makes the patients feel supported, to trust the physician’s judgment, and to engage with medical advice. Medical humanistic care aims to provide health care with honesty, empathy, compassion, altruism and respect to dignity and beliefs of the patients and their families [14, 15]. Communication skills were evaluated based on the doctor-patient communication (DPC) questionnaire and the ComOn check rating scale [16, 17]. As to humanistic care evaluation, we referred to the ICARE Scale [18].

Average GRS was determined and used to describe the overall performance quality. All rating scales were assessed by the assessors, and each performance received one of the five following grades: “excellent, satisfactory, borderline satisfactory, borderline fail and fail”.

### Debriefing

At the end of the scenario, a formal debriefing was conducted for the participants to reexamine the simulated case experience, share their mental model and foster the reasoning behind their clinical judgment. Debriefing is a crucial part in simulation training, which allows participants to gain a clear understanding of their actions and thoughts processes to promote learning outcomes and enhance future clinical performance.

### Post-simulation quiz

After completion of simulation activities and debriefing sessions, the participants completed another 10-question post-simulation quiz related to the diagnosis and treatment of asthma.

### Course survey

The students were invited to complete a course evaluation survey voluntarily at the end of the course, which contained questions focused on course evaluation, self-improvement, teaching model and equipment. The survey was based on the Kirkpatrick scale, with adjustments according to our case. The course evaluation was conducted on https://www.wjx.cn.

### Data analysis

Statistical analysis was performed with SPSS ver. 26.0. Pre- and post-simulation test scores, checklist, rating scales and survey responses were summarized by descriptive statistics. Data were expressed as mean ± standard deviation and analyzed by the Kolmogorov–Smirnov test (K-S test) to assess normality. The Mann–Whitney test was used to analyze differences between the two groups in pre-simulation test scores, post-simulation test scores, checklist and rating scales. The Wilcoxon's Sign Rank test was used to analyze the difference between the pre-test and post-test scores in each group. Spearman correlation coefficients were used to describe the associations of checklist items with rating scales. *P* < 0.01 was considered statistically significant.

## Results

### Characteristics of the study population

A total of 203 participants were included in this study. They finished diagnostics and internal medicine courses and had no prior experience of simulation education. Participant age ranged from 20 to 24 years. Totally 69.5% of the participating students were female, and 30.5% were male. Of all patients, 101 and 102 were assigned to the unexposed (not exposed to simulation) and pre-exposure (myocardial infarction simulation 2 weeks before asthma simulation) groups. Each group was divided into 25 subgroups of 4 to 5 students.

### Pre-simulation and post-simulation quiz scores in the unexposed and pre-exposure groups

Average pre- and post-simulation test scores in both groups are shown in Table 1. There were no differences in average scores between the unexposed and pre-exposure groups in pre-simulation test scores (77.35 ± 12.50 vs. 77.03 ± 12.45, *p* = 0.911) and post-simulation scores (88.33 ± 9.16 vs. 86.83 ± 8.80, *p* = 0.218).

**Table 1:** Average test scores in pre-simulation and post-simulation

Average test scores	Unexposed Group (*n* = 102)	Pre-exposure Group (*n* = 101)	p
Pre-test	77.35 ± 12.50	77.03 ± 12.45	0.911
Post-test	88.33 ± 9.16	86.83 ± 8.80	0.218
p	** < 0.001**	** < 0.001**	

Knowledge improved after the simulation, as demonstrated by post-simulation scores that were higher than pre-simulation scores in the unexposed (77.35 ± 12.50 vs. 88.33 ± 9.16, *p* < 0.001) and pre-exposure (77.03 ± 12.45 vs. 86.83 ± 8.80, *p* < 0.001) groups.

### Performance evaluation during the simulation

Performance scores are presented in Table 2. Checklist completion was improved in the pre-exposure group compared with the unexposed group (19.32 ± 1.50 vs. 17.6 ± 1.61, *p* < 0.001). Average GRS was also higher in the pre-exposure group than in the unexposed group (3.82 ± 0.34 vs. 3.44 ± 0.38, *p* = 0.001).

**Table 2:** Average performance score during scenario

Average performance score	Unexposed Group (25 groups)	Pre-exposure Group (25 groups)	p
Checklist completion	17.6 ± 1.61	19.32 ± 1.50	** < 0.001**
Average GRS	3.44 ± 0.38	3.82 ± 0.34	**0.001**
Communication skills	2.64 ± 0.64	3.24 ± 0.60	**0.002**
History taking	2.60 ± 0.71	3.16 ± 0.62	0.050
Physical examination	3.76 ± 0.52	4.20 ± 0.65	0.013
Auxiliary examination interpretation	4.12 ± 0.53	4.24 ± 0.52	0.425
Treatment	3.80 ± 0.71	4.04 ± 0.61	0.19
Humanistic care	2.60 ± 0.71	3.16 ± 0.62	**0.005**

To further investigate the abilities of these students, six individual rating scales were used in both groups. It was found that pre-exposure to simulation had no effects on medical history taking (2.60 ± 0.71 in the unexposed group vs. 3.16 ± 0.62 in the pre-exposure group, *p* = 0.050), physical examination (3.76 ± 0.52 in the unexposed group vs. 4.20 ± 0.65 in the pre-exposure group, *p* = 0.013), auxiliary examination interpretation (4.12 ± 0.53 in the unexposed group vs. 4.24 ± 0.52 in the pre-exposure group, *p* = 0.425) and treatment (3.80 ± 0.71 in the unexposed group vs. 4.04 ± 0.61 in the pre-exposure group, *p* = 0.19).

Interestingly, scores in communication skills (2.64 ± 0.64 in the unexposed group vs. 3.24 ± 0.60 in the pre-exposure group, *p* = 0.002) and medical humanistic care (2.60 ± 0.71 in the unexposed group vs. 3.16 ± 0.62 in the pre-exposure group, *p* = 0.005) were higher in the pre-exposure group compared with the unexposed group.

What’s more, we hypothesized that there are associations among those performance items. Spearman correlation coefficients were used to analyze the associations of checklist items with GRS (Table 3). Average GRS showed statistically significant positive associations with checklist completion, physical examination and treatment in both groups. Checklist completion was positively correlated with medical history taking in the unexposed group and humanistic care in the pre-exposure group. Checklist completion showed statistically significant positive correlations with medical history taking, physical examination and treatment only in the unexposed group. Treatment was also significantly and positively correlated with medical history taking and physical examination in the unexposed group. Medical history taking also showed a significant and positive correlation with physical examination in the unexposed group.

**Table 3:** Correlation among participants’ competencies

	Average GRS rho (p)	CC rho (p)	HT rho (p)	PE rho (p)	AEI rho (p)	TT rho (p)	HC rho (p)
**Unexposed Group**							
CC	0.777^*^ (< 0.001)						
HT	0.873^*^ (< 0.001)	0.775^*^ **(< 0.001)**					
PE	0.654^*^ (< 0.001)	0.599^*^ **(0.002)**	0.699^*^ **(< 0.001)**				
AEI	0.441 (0.027)	0.270 (0.192)	0.277 (0.180)	0.237 (0.253)			
TT	0.802^*^ (< 0.001)	0.678^*^ **(< 0.001)**	0.858^*^ **(< 0.001)**	0.530^*^ **(0.006)**	0.263 (0.205)		
HC	0.466 (0.019)	0.410 (0.042)	0.201 (0.335)	0.195 (0.351)	0.048 (0.819)	0.083 (0.694)	
CS	0.225 (0.279)	0 (0.999)	-0.016 (0.938)	-0.241 (0.245)	0.021 (0.921)	0.051 (0.808)	-0.065 (0.759)
**Pre-exposure Group**							
CC	**0.541**^**#**^ **(0.005)**						
HT	0.498 (0.011)	0.344 (0.092)					
PE	**0.761**^**#**^** (< 0.001)**	0.258 (0.214)	0.200 (0.337)				
AEI	0.337 (0.1)	0.172 (0.412)	-0.072 (0.731)	0.361 (0.076)			
TT	**0.688**^**#**^** (< 0.001)**	0.274 (0.186)	0.476 (0.016)	0.375 (0.065)	-0.035 (0.869)		
HC	**0.532**^**#**^** (0.006)**	0.358 (0.079)	0.055 (0.795)	0.339 (0.097)	0.123 (0.557)	0.192 (0.357)	
CS	0.457 (0.022)	0.323 (0.116)	0.068 (0.747)	0.292 (0.156)	-0.057 (0.786)	0.301 (0.144)	-0.041 (0.845)

^*^p < 0.001 in unexposed group represented that there is a positive correlation between the corresponding two abilities in the table

^**#**^*p* < 0.001 in pre-exposure group represented that there is a positive correlation between the corresponding two abilities in the table

*GRS* Global Rating Scale, *CC* Checklist Completion, *HT* History Taking, *PE* Physical Examination, *AEI* Auxiliary Examination Interpretation, *TT* Treatment, *HC* Humanistic Care, *CS* Communication Skills

### Course evaluation survey after simulation training

A total of 106 students (52.21%) completed the post-course survey (Table 4). Large percentages of students, 73.21% and 26.13% strongly agreed and agreed, respectively, that SBT improved their skills in problem-solving, decision-making and effective communication with patients, and resulted in a comprehensive understanding of their strengths and weaknesses in the management of asthma exacerbation. In addition, the student could perform unlimited repetitions on the simulator without any risk and receive immediate feedback according from the instructor. However, 0.66% of participants indicated that the training undermined their confidence and increased the tension and fear in facing clinical problems.

**Table 4:** Course evaluation survey

10 items to evaluate this course	Strongly disagree	Disagree	Agree	Strongly agree	Total students
1. The training improved confidence in facing clinical problems	-	**3**	23	80	106
2. The training eased tension and fear in facing clinical issues	-	**4**	28	74	106
3. The training improved my ability to recognize and deal similar situations	-	-	25	81	106
4. The training deepen my understanding of clinical knowledge and the ability of clinical practice	-	-	27	79	106
5. The training provided an opportunity for teamwork	-	-	30	76	106
6. During the simulation, I discussed the needs of patients with the team through effective communication skills	-	-	30	76	106
7. Self-summary and self-feedback helped to recognize own strengths, weaknesses and blind spots	-	-	36	70	106
8. The instructor’s feedback were helpful	-	-	33	73	106
9. The equipment was realistic and helpful	-	-	21	85	106
10. The training was very valuable	-	-	24	82	106
Average student number	0	0.7	27.7	77.6	106
Percentage(Average/Total)	0	0.66%	26.13%	73.21%	100%

## Discussion

This study compared the performance of two student groups that underwent asthma exacerbation simulation training, including the pre-exposure and unexposed groups, to assess how a prior experience in simulation training affects performance in subsequent an asthma exacerbation simulation.

The above results revealed that previous exposure to SBT was helpful in subsequent SBT in communication skills with patient and medical humanistic care. Medical humanistic care always runs through the whole process of communication with patients, especially in critically ill, elderly, pediatric and tumor patients [14, 15, 19, 20]. As for communication skills, our assessors mainly focused on the following aspects: whether the student used simple and concise language to the family of simulated patient, whether the student summarized what the patient’s family said all the way through, and how the student explained the poor prognosis of simulated patient to the family. In terms of humanistic medical care, the assessors observed whether the student emphasized the psychological and social aspects of medical care, and whether the student respected the patients’ family and listened to them, etc. This study demonstrated that the pre-exposure group worked better compared with the unexposed group, which verified that practice results in improvement [21, 22].

Moreover, we also found that medical students previously exposed to simulation training analyze asthma exacerbation cases more comprehensively. We set 6 domain and 22 items in the checklist; in the pre-exposure group, participants completed more items in the checklist in the subsequent course. Asthma exacerbation is frequently encountered in respiratory medicine clinic and emergency department. Failure to recognize the signs of patient deterioration in time could lead to a fatal outcome. Therefore, familiarity with the identification and management of asthma exacerbation immediately is a necessary skill for medical students. The more items the student completes in the checklist, the higher the odds of treatment success for the simulated patient.

In addition, this study corroborated other reports. Professional knowledge improved after the simulation, with the participants’ scores being higher than values before simulation [9]. In a previous report, students who practiced with task variation did not outperform those who practiced repeatedly on the same task; in this study experience in myocardial infarction had no significant influence on knowledge improvement in asthma scenario [10, 23]. Similar mistake and de-normalization occurred in medical history taking, physical examination, auxiliary examination interpretation and treatment in both groups. Any neglected items during the simulation were listed one-by-one at the end of the debriefing, discussing how to prevent them in the future.

In the course evaluation survey, you might note that 0.66% of students find the experience of SBT stressful. For these students who need extra assistance, educators might consider offering additional debriefing and practice opportunities to help them benefit from SBT.

There were several limitations in this study. Firstly, the students were grouped into the pre-exposure and unexposed groups by student number, without complete randomization. Secondly, we acknowledged the possibility that participants discussed the course objectives with their classmates, despite a request to keep their experience confidential for the sake of the research study.

In conclusion, this study comprehensively assessed the effects of prior exposure to simulation training on performance in subsequent simulation-based asthma exacerbation training. The results are encouraging, suggesting that pre-exposure to SBT could improve communication skills, medical humanistic care, as well as checklist completion in medical students. This study adds to the accumulating literature on SBT, demonstrating the particular effects in specific learning domains of prior SBT exposure on subsequent experiences, even on non technical skills. These findings may prove useful for future researchers and educators designing simulation-based training experiments.

## Supplementary Information

**Additional file 1.**

**Additional file 2.**

## Data Availability

All data generated or analyzed during this study are included in this published article.

## Abbreviations

SBT: Simulation-based training
GRS: Global rating scale
