# Supplementary material for: Simulation-based training in asthma exacerbation for medical students: effect of prior exposure to simulation training on performance
Source: BMC Med Educ. 2022 Mar 31;22:223. doi: 10.1186/s12909-022-03300-2 (PMC8973632; doi:10.1186/s12909-022-03300-2)
Supplement: Supplementary file 1 — Additional file 1. [file 12909_2022_3300_MOESM1_ESM.docx]

Supplementary Figure

**
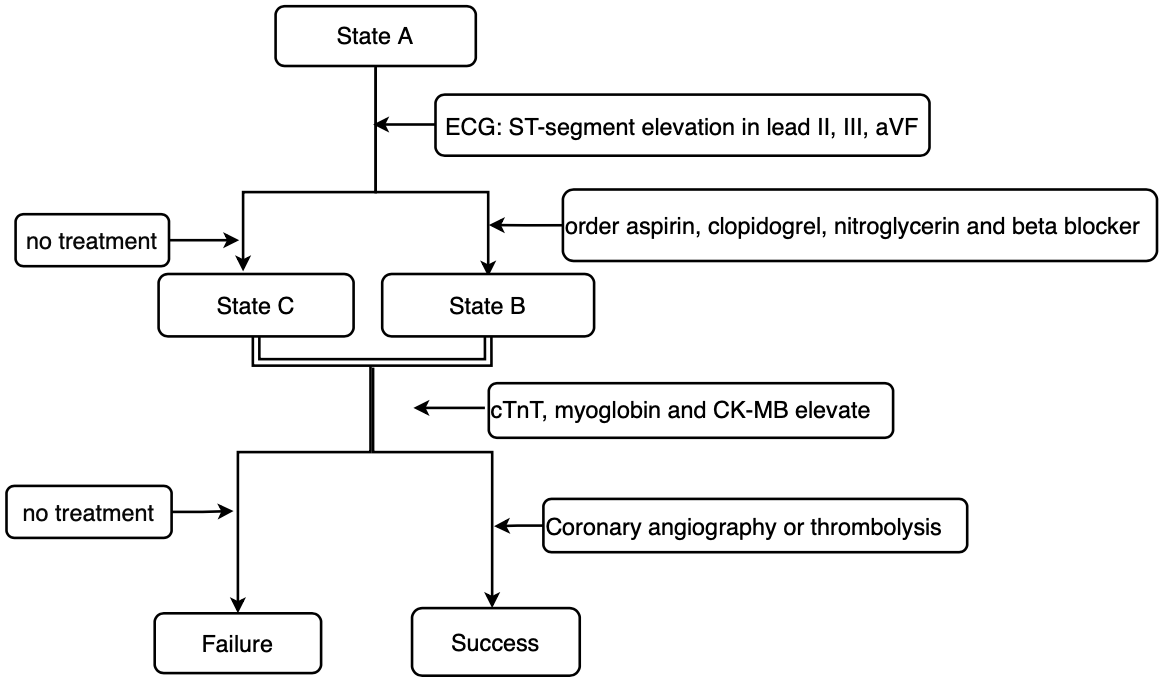
Figure 1 Scenario flow chart of asthma exacerbation**

Annotations: A, B and C present states of simulated patient.

**State A**: Conscious, pressure or crushing chest pain associated with shortness of breath, nausea/vomiting or diaphoresis, HR 110bpm, RR 30bpb, Bp145/90mmHg, Oxygen saturation (SaO2) 91%

**State B**: Conscious, chest pain gets a little better than before. HR 100bpm, RR 25bpb, Bp126/85mmHg, SaO2 92%

**State C**: Exacerbated dyspnea and chest pain, feeling impending death, HR 130bpm, RR 33bpb, Bp162/98mmHg, SaO2 85%
