# Supplementary material for: Simulation-based training in asthma exacerbation for medical students: effect of prior exposure to simulation training on performance
Source: BMC Med Educ. 2022 Mar 31;22:223. doi: 10.1186/s12909-022-03300-2 (PMC8973632; doi:10.1186/s12909-022-03300-2)
Supplement: Supplementary file 2 — Additional file 2. [file 12909_2022_3300_MOESM2_ESM.docx]

Supplementary Table

Table 1 Checklist of asthma exacerbation simulation course

| Major domain | Items (total 22 items) | Yes | No |
| --- | --- | --- | --- |
| **History taking** | Identify the onset time |  |  |
|  | Identify the possible cause |  |  |
|  | Check the chief complaints (including dyspnea, wheezing, etc ) |  |  |
|  | Identify the patient’s past medical history (including history of similar episodes) |  |  |
|  | Identify personal history (including allergic history, medication history, diet history) |  |  |
|  | Identify family history |  |  |
| **Physical examination** | Check thoracic shape |  |  |
|  | Auscultate breath sounds |  |  |
|  | Check wheezing sounds |  |  |
|  | Auscultate heart sounds |  |  |
|  | Check respiratory rate |  |  |
|  | Check SpO2 |  |  |
|  | Check heart rate and blood pressure |  |  |
| **Observation of disease development** | Notice changes in consciousness |  |  |
|  | Notice changes in vital signs |  |  |
|  | Notice changes in breath sounds |  |  |
| **Auxiliary examination** | Take arterial blood gas analysis |  |  |
|  | Inspect X-ray, ECG, myocardiac enzyme, D-dimer and Brain natriuretic peptide (BNP) |  |  |
| **Treatment** | Provide appropriate concentration of oxygen inhalation |  |  |
|  | Check airway secretion when patient’s condition worsens |  |  |
|  | Re-check patient’s vital sign and arterial blood gas |  |  |
| **Diagnosis** | Diagnosis as “asthma exacerbation” |  |  |
| Total |  |  |  |
